# Use of Calcium Chlorid to Prevent Hemorrhage after Tooth Extraction

**Published:** 1902-11-15

**Authors:** 


### USE OF CALCIUM CHLORID TO PREVENT HEMORRHAGE AFTER TOOTH-EXTRACTION.

As is well known, the extraction of a tooth may give rise to severe hemorrhage in persons suffering from hemophilia. Dr. C. E. Vallis, assistant dental surgeon in King's College Hospital, London, has observed the case of a woman aged twenty-five, presenting the hemorrhagic diathesis, in whom the extraction of tooth was followed by a hemorrhage which lasted thirty-six hours. As the teeth of this patient were in very bad condition, and as the extraction of all the carious teeth became necessary because of dyspeptic troubles from which the patient was suffering, Dr. Vallis endeavored to use some means by which the coagulability of the blood would be increased. With that object in view he administered calcium chlorid in weak doses during a period of eight days previous to the time set for the performance of the operation. He extracted an incisor, an operation that was performed without the slightest loss of blood. Continuing to administer the same agent, he was able to extract every tooth without hemorrhage. Since then, Dr. Vallis has observed a similar case in which calcium chlorid has given the same satisfactory results. The disagreeable taste of this medicament, and the slight tendency to constipation which it induces, are the only inconveniences connected with its administration, even after continuous use during a period of three to four weeks.—*Cosmos*, extract from *Stomatologic Revue*, of Paris.

